# Combined Experimental and Theoretical Investigation into the Photophysical Properties of Halogenated Coelenteramide Analogs

**DOI:** 10.3390/molecules27248875

**Published:** 2022-12-14

**Authors:** Ana Carolina P. Afonso, Patricia González-Berdullas, Joaquim C. G. Esteves da Silva, Luís Pinto da Silva

**Affiliations:** 1 Chemistry Research Unit (CIQUP), Institute of Molecular Sciences (IMS), Department of Geosciences, Environment and Territorial Planning, Faculty of Sciences, University of Porto, R. Campo Alegre s/n, 4169-007 Porto, Portugal; 2 LACOMEPHI, GreenUPorto, Department of Geosciences, Environment and Territorial Planning, Faculty of Sciences, University of Porto, R. Campo Alegre s/n, 4169-007 Porto, Portugal

**Keywords:** chemiluminescence, bioluminescence, Coelenterazine, Coelenteramide, fluorescence, photophysics, microenvironment probe, heavy-atom effect

## Abstract

Marine Coelenterazine is one of the most well-known chemi-/bioluminescent systems, and in which reaction the chemi-/bioluminophore (Coelenteramide) is generated and chemiexcited to singlet excited states (leading to light emission). Recent studies have shown that the bromination of compounds associated with the marine Coelenterazine system can provide them with new properties, such as anticancer activity and enhanced emission. Given this, our objective is to characterize the photophysical properties of a previously reported brominated Coelenteramide analog, by employing a combined experimental and theoretical approach. To better analyze the potential halogen effect, we have also synthesized and characterized, for the first time, two new fluorinated and chlorinated Coelenteramide analogs. These compounds show similar emission spectra in aqueous solution, but with different fluorescence quantum yields, in a trend that can be correlated with the heavy-atom effect (F > Cl > Br). A blue shift in emission in other solvents is also verified with the F–Cl–Br trend. More relevantly, the fluorescence quantum yield of the brominated analog is particularly sensitive to changes in solvent, which indicates that this compound has potential use as a microenvironment fluorescence probe. Theoretical calculations indicate that the observed excited state transitions result from local excitations involving the pyrazine ring. The obtained information should be useful for the further exploration of halogenated Coelenteramides and their luminescent properties.

## 1. Introduction

Chemiluminescence (CL) consists in the emission of radiation due to a chemical reaction [1,2,3,4]. A sub-type of CL is bioluminescence (BL), in which light is emitted due to a biochemical reaction (involving an enzyme or photoprotein) [1,2,3,4]. BL is widespread in nature and can be found in organisms as different as fireflies, jellyfishes, bacteria, and fungi, among others [1,2,3,4]. Typically, light emission from CL/BL reactions originates due to the formation of a high-energy peroxide intermediate, which decomposes rather quickly with high exothermicity, which allows for chemiexcitation to excited states [5,6,7,8,9,10].

Both CL and BL reactions present a diminished probability for autofluorescence arising from the background signal, which increases the signal-to-noise ratio, as they do not require photoexcitation to generate the chemiexcited light emitter [11,12]. Given this, CL/BL can generate luminescent signals with high sensitivity and almost no background noise [13]. This feature is particularly useful for applications in biologic media, which explains why CL/BL systems have been gaining practical applications in fields such as cancer therapy [14,15,16], real-time imaging [17,18,19] and (bio)sensing [20,21,22,23,24].

**Figure 1:**
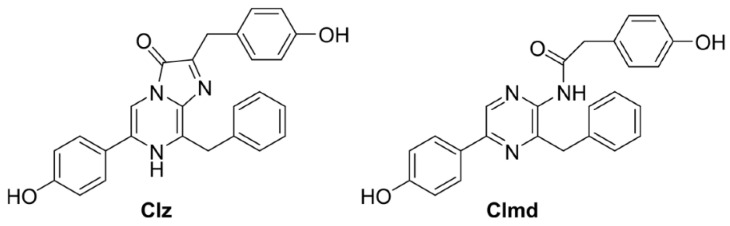
Chemical structures of native Clz and Clmd.

It should be noted that around 80% of all luminescent organisms are present in the oceans, and most of them employ imidazopyrazinone-based compounds as BL substrates [25], such as Coelenterazine (Clz, Figure 1). In fact, Clz is one of the most well-known and studied compounds among the existing CL and/or BL substrates [26,27,28,29,30]. Interestingly, Clz is capable of both BL (when in the presence of either photoproteins or luciferase enzymes) [2,4] and CL (when in polar aprotic solvents, such as DMF or DMSO, or in the presence of reactive oxygen species, such as superoxide anion) [31,32,33]. Irrespective of this, CL/BL reactions of Clz occur via the same general mechanism [2,4,26,27,28,29,30,31,32,33]: there is the oxygenation of the imidazopyrazinone core, with the formation of a high-energy peroxide intermediate; this latter compound is highly unstable and undergoes decomposition almost instantly. During this reaction, the reacting molecules can cross to the singlet excited state, thereby generating the chemiexcited light emitter Coelenteramide (Clmd, Figure 1). Clmd is then the species that emits light during both CL and BL reactions and possesses an amidopyrazine core (instead of the imidazopyrazinone core of Clz) [34,35,36].

It should be noted that besides developing practical applications for native Clz (and other imidazopyrazinones), the research community has also been active in the development of new molecules based on Clz and with enhanced features, such as red-shifted emission, brighter light emission, and a longer emission half-life [37,38,39,40]. Two of the most well-known examples are commercial Coelenterazine 400a (Clz400a) [37,38] and Coelenterazine-e (Clz-e) [41].

Our group has also been active in the development of novel Clz analogs with both enhanced and new properties [42,43,44,45,46,47,48]. This effort has had a focus on the introduction of bromine (Br) heteroatoms into the imidazopyrazinone scaffold of Clz (either directly into the core, or by being part of different functional groups). Quite interestingly, this type of modification has provided some novel analogs with quite enhanced CL emission in aqueous solution when compared with native Clz [46,47,48]. For others, the introduction of Br has provided them with anticancer activity toward different cancer cell types (prostate, breast, neuroblastoma, lung, and/or gastric cancer). Thus, it is clear that bromination is a relevant strategy in modifying Clz.

**Figure 2:**
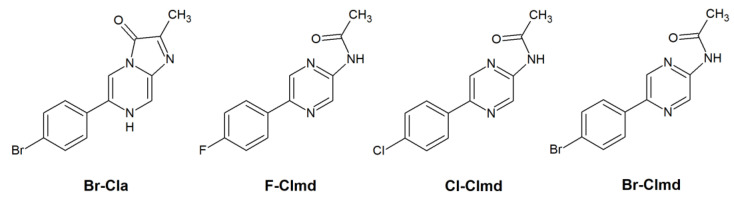
Chemical structures of the halogenated Br-Cla and Clmds (F-, Cl-, and Br-Clmd).

Among the developed analogs, we highlight one in which the phenol, benzyl, and *p*-cresol moieties of Clz (Figure 1) are replaced by a bromophenyl moiety, a hydrogen atom, and a methyl group (Br-Cla, Figure 2), respectively [43,44,45]. This compound presents cytotoxicity toward both prostate and breast cancer (IC_50_ of 24.28 and 21.56 μM, respectively), while analysis with non-cancer cells demonstrated a relevant profile of tumor selectivity [43,44]. Quite interestingly, we have found that its corresponding Clmd version (Br-Clmd, Figure 2) also presents anticancer activity, albeit apparently not by the same mode of action [45]. More specifically, Br-Clmd showed activity toward both gastric and lung cancer (IC_50_ of 16.2 and 10.1 μM, respectively) [45]. Given this, it does appear that the modification of Clmd with the inclusion of Br heteroatoms is also a good strategy to tune the properties of this species.

Thus, given the previous information and the role of Clmd as a light emitter in CL/BL reactions [34,35,36], the aim of this work is then to evaluate, for the first time, the photophysical properties of Br-Clmd (Figure 2) by employing a combined experimental and theoretical approach. With this study, we intend to assess whether bromination is a relevant strategy to improve/modify the luminescent properties of Clmd-based compounds. To further evaluate whether changes are indeed due to Br, and not due to more general halogen-based effects, we have also synthesized and studied two novel Clmd analogs: F- and Cl-Clmd (Figure 2). The data obtained in this study should be useful for researchers focused on the development of Clz/Clmd-based systems with enhanced/new features.

## 2. Results and Discussion

### 2.1. Photophysical Characterization of the Clmd Analogs

**Figure 3:**
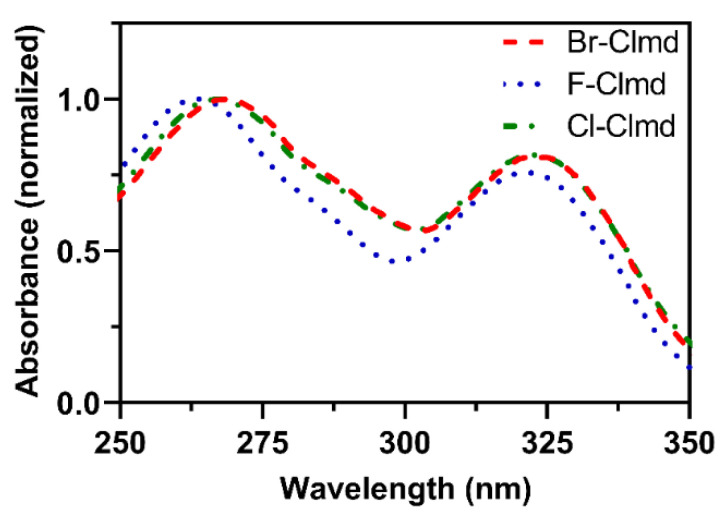
UV–Vis spectra of the halogenated Clmds in aqueous solution.

The absorption spectra of the three halogenated Clmd analogs, in aqueous solution, are presented in Figure 3. The three analogs present spectra with a very similar shape and peak position. This is particularly true for Cl- and Br-Clmd, whose spectra are composed of two peaks at ~270 and ~320 nm, with identical relative intensities between them. The absorption spectrum of F-Clmd is also similar, as it is composed of two peaks, with one of them with a maximum also at ~320 nm. However, the other peak is slightly blue-shifted (~260 nm). Moreover, it appears that the relative difference in intensity between the peaks for F-Clmd is not the same as for Cl-/Br-Clmd. Compared with the literature [36,49], the absorption of all halogenated compounds (red-shifted peak at ~320) is blue-shifted relative to native Clmd (335–340 nm).

**Figure 4:**
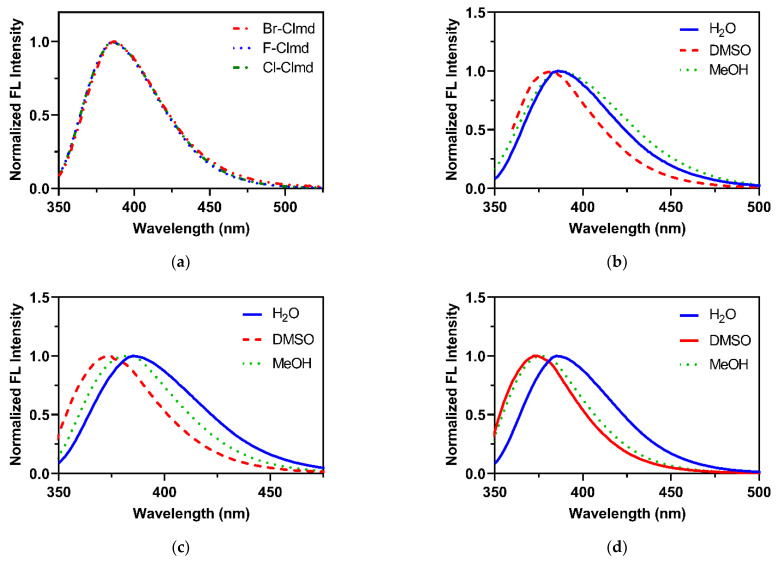
Normalized fluorescence (FL) spectra for the compounds studied in different solvents. (**a**) Halogenated Clmds in deionized water; (**b**) Br-Clmd; (**c**) F-Clmd; (**d**) Cl-Clmd. Excitation at 310 nm.

The fluorescence spectra of the compounds were obtained in different solvents (Figure 4): deionized water, dimethyl sulfoxide (DMSO), and methanol (MeOH). These solvents are typically used in the study of the CL/BL system of Clz/Clmd [12,27,28,31,32,33,35,46,47].

In an aqueous solution (Figure 4a), the three halogenated compounds present overlapped spectra, with an emission maximum at 385 nm. Thus, in aqueous solution, varying the halogen heteroatom does not affect the shape of the fluorescence spectra of these compounds. This emission maximum is in line with the emission typically attributed to the neutral form of native Clmd, which emits from 386 to 391 nm in benzene [49] and up to 420 nm in MeOH [35]. This information helps us to attribute the emitted fluorescence to the neutral form (Figure 2) of F-/Cl-/Br-Clmd.

The measurement of fluorescence in other solvents (Figure 4) indicates that the more red-shifted emission is found in water, while the more blue-shifted emission was measured in DMSO for all compounds (by 10 nm). Interestingly, the main difference between the compounds is their fluorescence in MeOH. For Br-Clmd, there is an overlap between the emission spectra in aqueous solution and in MeOH. However, for Cl-Clmd, there is a ~5 nm blue shift between the emission in water and in MeOH. This blue shift further increases to ~8 nm for F-Clmd. Thus, there does appear to exist a halogen-dependent effect regarding the emission of the compounds in MeOH.

Finally, given the highest emission wavelength for these compounds (385 nm) and the emission maxima previously reported for native Clmd (up to 420 nm [35,49]), it is clear that the emission of these Clmd analogs is more blue-shifted than for the natural compound.

### 2.2. Fluorescence Quantum Yield

The QY values for the three studied compounds, in the studied solvents, are presented in Table 1. There is great variability for the three compounds in the aqueous solution, as the QY values range from 8 to 26%. Interestingly, we can see that the QY (in water) increases from the heaviest halogen to the lightest one: Br (8%) < Cl (23%) < F (26%). This could be attributed to the heavy-atom effect [50], as it can reduce QY by enhancing intersystem crossing (ISC) to triplet states [44,50]. In fact, the introduction of Br heteroatoms is a typical strategy to enhance ISC, due to the heavy-atom effect, which correlates well with the obtained results in aqueous solution.

However, this halogen-dependent effect was no longer observed in DMSO, as the QY values were similar for all three compounds. It should be noted, nevertheless, that this similarity results from a relevant decrease in yield (from 23–26% to 12–14%) for F-/Cl-Clmd, and an increase for Br-Clmd (from 8 to 14%). In MeOH, there is an even higher increase to 17%, regarding its yield in an aqueous solution. For F-/Cl-Clmd, their QY values in MeOH were higher than in DMSO, but lower than in water. For these organic solvents, there is not a particularly noticeable heavy-atom effect, as the highest QY was observed for Cl-Clmd, followed by Br-Clmd and then by F-Clmd.

In short, there is an indication that the heavy-atom effect plays a role in the QY of the halogenated Clmd analogs in aqueous solution. However, in other solvents, other factors besides the heavy-atom effect should have a greater role on the obtained QY values. Nevertheless, if the reduced QY values in aqueous solutions (closer to biological media) are indeed due to the heavy-atom effect, this could mean that Br-Clmd could have intrinsic value as the basis for a photosensitizer [50].

### 2.3. Fluorescence Response to Variations in Solution

There is an increasing focus among the research community to develop fluorescent probes for microenvironment-related parameters (such as polarity, viscosity, and pH) [51,52,53]. These play important roles in the control of the physical–chemical behaviors of local molecules [51,52,53]. Thus, such probes can be very useful in the study of both physiological and pathological processes [51,52,53].

**Figure 5:**
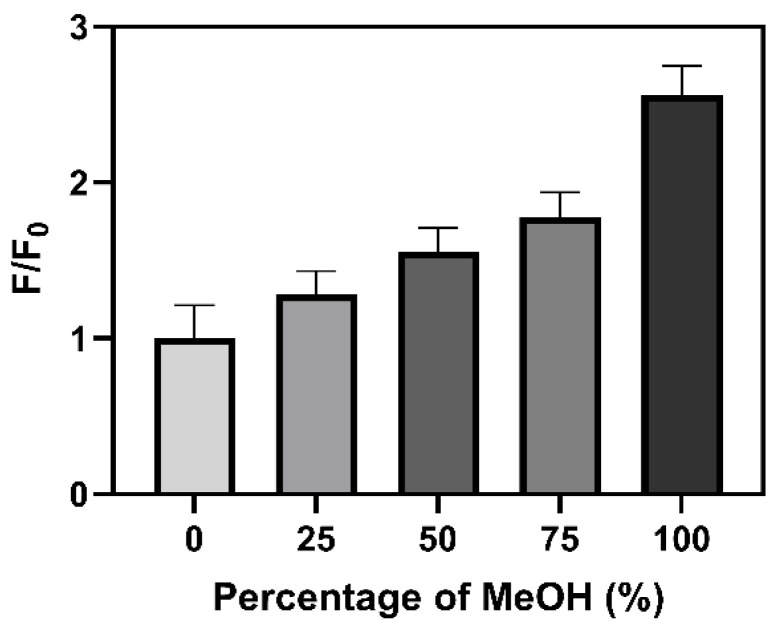
Variation in Br-Clmd fluorescence intensity (F/F_0_) in mixtures of water and MeOH (from 0 to 100%).

As can be seen in both Table 1 and Figure 4, while the emission wavelength of Br-Clmd is not particularly affected by the microenvironment, this is not the case regarding its QY. Thus, it is possible that this molecule could have potential to be used as a fluorescent probe for the local microenvironment by measuring the variation in its fluorescent intensity. To better assess this, we then measured the fluorescence intensity (F/F_0_) of a 10 μM solution of Br-Clmd with an increasing ratio of MeOH in water (from 0 to 100%, with increments of 25%). The results can be found in Figure 5. We did observe a gradual increase in fluorescence with an increasing ratio of MeOH in the solution, reaching an approximately 2.5 times increase in pure MeOH and almost two times in 25%/75% water/MeOH. Thus, the fluorescence of Br-Clmd is indeed affected by changes in the microenvironment. Given this, this type of compound has potential to be further explored as a basis for new probes for microenvironment-related parameters. Regarding the reason that this change in fluorescence intensity occurs, it should be noted that it has been previously reported that water can act as a fluorescence quencher [54,55,56]. In fact, different fluorophores have been found to present lower QY values in water than in organic solvents [55,56]. Therefore, the quenching effect of water can help to explain the intensity variation here observed. Nevertheless, further research should be performed in the future to better understand this phenomenon.

### 2.4. Theoretical Investigation of the Photophysics of Br-Clmd

**Figure 6:**
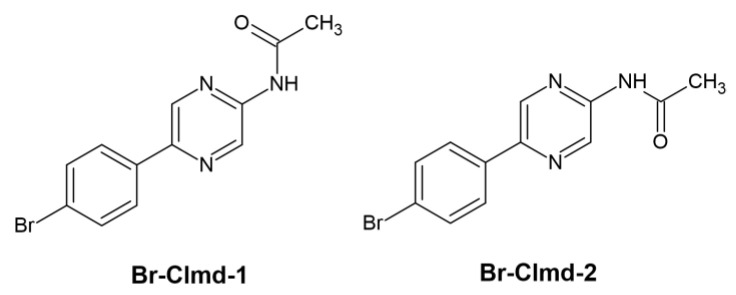
Possible conformations for Br-Clmd.

To obtain further information about the photophysics of Br-Clmd, we attempted to characterize the electron excitation of this molecule in implicit water (with a vertical approximation) by using hole–electron analysis [57]. This was performed at the TD-DFT level of theory, with three different density functionals: ωB97XD, CAM-B3LYP, and PBE0. We focused on the neutral species of Br-Clmd, given the match between the photophysical properties here measured with those of native Clmd [35,49]. Furthermore, Br-Clmd can potentially coexist in one of two conformations (Br-Clmd-1 and Br-Clmd-2), as seen in Figure 6. The latter one was found be more stable than the former by 4.2 kcal mol^−1^ (Gibbs free energy with thermal corrections), and so we focused on Br-Clmd-2 in the subsequent analysis.

In Table 2, we present the excitation wavelength (λ_ex_, in nm), the oscillator strength (*f*), and the S_r_ index for the *S*_0_ → *S*_1_ vertical excitation of Br-Clmd-2, as calculated with the three density functionals: ωB97XD, CAM-B3LYP, and PBE0. The S_r_ index characterizes the overlapping extent of holes and electrons (its theoretical upper limit being 1.0) [57]. While PBE0 does provide λ_ex_ values close to the experimentally determined ones (Figure 3), ωB97XD and CAM-B3LYP do not appear to reproduce experiment well. Nevertheless, all agree on the value for the S_r_ index: 0.745–0.772. It should be remembered that this index evaluates the overlapping extent of hole and electron distribution (upon electron excitation) and possesses a theoretical upper limit of 1.0 [57]. This indicates that more than half of the hole and electron are perfectly matched [57]. Thus, we can attribute this *S*_0_ → *S*_1_ transition to the LE type.

**Figure 7:**
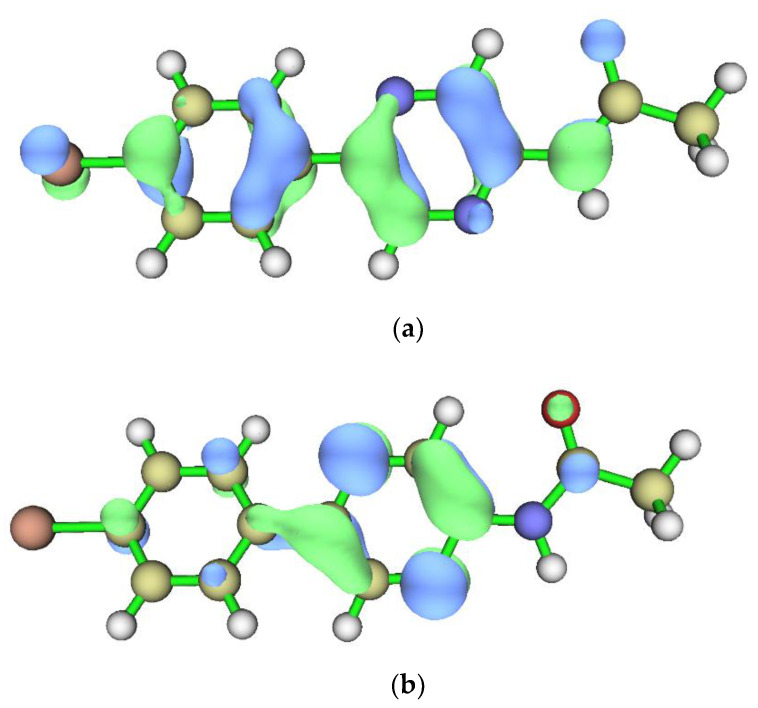
HOMO (**a**) and LUMO (**b**) orbitals for Br-Clmd-2 in implicit water, when calculated at the TD-PBE0 level of theory.

It was also found that the *S*_0_ → *S*_1_ transition corresponds to a HOMO → LUMO excitation (Figure 7). More specifically, the studied transition appears to be a π → π * local excitation (LE), with a relevant overlap of hole and electron in the pyrazine ring.

## 3. Materials and Methods

### 3.1. Synthesis of Halogenated Clmds

The synthesis of the studied compounds started with the functionalization of commercial 5-bromopyrazin-2-amine, via a Suzuki–Miyaura cross-coupling reaction with commercial boronic acids (with procedures described in more detail in the Supplementary Materials). This yielded the corresponding F-, Cl-, and Br-substituted phenylpyrazin-2-amine (Coelenteramine, Clm) synthesis intermediates, which were already described in [43,44,45,47]. The final Clmd structures were obtained for all three compounds through N-acetylation of the Clm intermediates, by using pyridine as the base to avoid the formation of the disubstituted subproduct. The structural characterization was performed by using both ^1^H- and ^13^C-NMR spectroscopy, as well as FT-MS spectromety. Br-Clmd have already been described in the literature [45], while further details for F- and Cl-Clmd can be found in the Supplementary Materials (Figures S1–S4).

### 3.2. Photophysical Characterization

The fluorescence spectra were analyzed via fluorescence spectroscopy, using a standard 10 mm quartz cuvette, with a Horiba Yvon Fluoromax-4 fluorimeter [58]. The emission and excitation spectra were obtained with a 1 nm capture interval and 2 nm slit width. Absorption spectra were obtained with a VWRs UV-3100PC spectrophotometer, by using quartz cells. Assays were performed with a concentration of 10 μM of the studied compounds.

### 3.3. Determination of the Fluorescence Quantum Yield

The fluorescence quantum yield (QY) was calculated by comparing the integrated luminescence intensities and the absorbance values of the compounds with the following equation:

(1) $$QY=QY_{R}\times \frac{Grad}{Grad_{R}}\times \frac{\eta^{2}}{\eta_{R}^{2}}$$

In the equation, *QY* is the fluorescence quantum yield, *Grad* is the gradient from the plot of integrated fluorescence intensity versus absorbance, and *η* is the refractive index. The subscript *R* refers to the reference fluorophore with a known *QY*. In this work, quinine sulfate in 0.1 M H_2_SO_4_ was used, with a *QY* of 54% [59]. Quinine sulfate was the fluorescence standard selected as it has a similar excitation wavelength and emission spectrum to the studied compounds [59]. The refractive index is 1.33 for aqueous solutions, 1.326 for methanol, and 1.479 for DMSO [60,61,62].

### 3.4. Theoretical Calculations

The geometry optimizations for the singlet ground state (*S*_0_) of the studied molecules were performed with the ωB97XD density functional [63]. A 6-31G(d,p) basis set was used for H, C, N, and O, while the LANL2DZ basis set was used for Br. Frequency calculations were performed at the same level of theory. The *S*_0_ energies were re-evaluated using single-point calculations with the same functional as before, while increasing the basis sets: 6-31+G(d,p) for H, C, N, and O, and LANL2DZ with polarization and diffuse functions for Br. The vertical excitations to singlet excited states were calculated at the TD-ωB97XD level of theory, with the previously mentioned basis sets. ωB97XD was chosen as it generally provides accurate estimates for π -> π * and *n* -> π * LE, charge transfer, and Rydberg states [64]. To limit density-functional-related errors, vertical excitations were also calculated with other functionals: CAM-B3LYP [65] and PBE0 [66]. All calculations were performed in an implicit solvent, by using a polarizable continuum model (IEFPCM). These calculations were performed by using the Gaussian 09 program package [67].

The electron excitation analysis was performed with the MultiWFN software [57], based on the Gaussian-09-based calculations. More specifically, the quantitative characterization of the hole and electron distribution (upon electron excitation) was performed by calculating the S_r_ index, which characterizes the overlapping extent of holes and electrons (its theoretical upper limit being 1.0) [57].

## 4. Conclusions

In this study, we investigated the photophysical properties of three halogenated Clmd analogs: F-Clmd, Cl-Clmd, and Br-Clmd. This investigation was performed with a combined experimental and theoretical approach. The measured UV–Vis and fluorescence spectra of the compounds were quite similar in aqueous solution, with absorption and emission at ~320 and 385 nm, respectively. These data indicate that the luminescence of the halogenated analogs is blue-shifted with respect to native Clmd. Interestingly, the emission of the analogs is blue-shifted in organic solvents, whose magnitude is related to the F–Cl–Br trend. Furthermore, the fluorescence quantum yield of the analogs in aqueous solution increases in the order of Br < Cl < F, which can be correlated with the heavy-atom effect (and possible enhancement of intersystem crossing). Of additional relevance is the fact that while the emission spectra of Br-Clmd is similar in different solvents, its fluorescence quantum yield changes significantly. In fact, changing the water/methanol ratio of mixtures led to an increase in the fluorescence intensity of this compound by around 2.5 times. Thus, this analog shows some potential for use as the basis for the development of a fluorescence probe to detect changes in the local microenvironment. Theoretical calculations at the TD-DFT level indicated that the excited state transitions here observed are local excitations involving mainly the pyrazine ring of Clmd species.

## Figures and Tables

**Table 1:** Fluorescence quantum yields (QY, in %) in different solvents.

	Br-Clmd	Cl-Clmd	F-Clmd
Water	8%	23%	26%
DMSO	14%	14%	12%
MeOH	17%	18%	15%

**Table 2:** Excitation wavelength (λ_ex_, in nm), oscillator strength (*f*), and S_r_ index for the *S*_0_ → *S*_1_ excitation of Br-Clmd-2 in implicit water, when calculated at the TD-DFT level of theory with different functionals.

Density Functionals	λ_ex_	*f*	S_r_
ωB97XD	286	0.71	0.772
CAM-B3LYP	288	0.72	0.769
PBE0	305	0.67	0.745

## Supplementary Materials

The following supporting information can be downloaded at: https://www.mdpi.com/article/10.3390/molecules27248875/s1, Figure S1: ^1^H-NMR, ^13^C-NMR and DEPT spectra of *N*-(5-(4-fluorophenyl)pyrazine-2-yl)acetamide (F-Clmd). Figure S2: ^1^H-NMR, ^13^C-NMR and DEPT spectra of *N*-(5-(4-chlorophenyl)pyrazin-2-yl)acetamide (Cl-Clmd). Figure S3: FTMS-ESI (+) spectrum of *N*-(5-(4-fluorophenyl)pyrazin-2-yl)acetamide (F-Clmd). Figure S4: FTMS-ESI (+) spectrum of *N*-(5-(4-chlorophenyl)pyrazin-2-yl)acetamide (Cl-Clmd).
