# Supplementary material for: Combined Experimental and Theoretical Investigation into the Photophysical Properties of Halogenated Coelenteramide Analogs
Source: Molecules. 2022 Dec 14;27(24):8875. doi: 10.3390/molecules27248875 (PMC9781228; doi:10.3390/molecules27248875)
Supplement: Supplementary file 1 [file molecules-27-08875-s001.zip › molecules-2066912-supplementary.pdf]

Supplementary Information for

Combined Experimental and Theoretical Investigation into the Photophysical Properties of Halogenated Coelenteramide Analogs

Table of Contents

1. General Synthetic Procedures S3

1.1. *N*-(5-(4-Fluorophenyl)pyrazin-2-yl)acetamide (**F-Clmd**) S3

1.2. *N*-(5-(4-Chlorophenyl)pyrazin-2-yl)acetamide (**Cl-Clmd**) S4

2. Supporting Figures S5

Figure S1. ^{1}H-NMR, ^{13}C-NMR, and DEPT spectra of *N*-(5-(4-fluorophenyl)pyrazin-2-yl)acetamide (**F-Clmd**)........................ S5

Figure S2. ^{1}H-NMR, ^{13}C-NMR, and DEPT spectra of *N*-(5-(4-chlorophenyl)pyrazin-2-yl)acetamide (**Cl-Clmd**) S6

Figure S3. FTMS-ESI (+) spectrum of *N*-(5-(4-fluorophenyl)pyrazin-2-yl)acetamide (**F-Clmd**) S7

Figure S4. FTMS-ESI (+) spectrum of *N*-(5-(4-chlorophenyl)pyrazin-2-yl)acetamide (**Cl-Clmd**)**........................ S7**

1. General Synthetic Procedures

Reagents and solvents were purchased from Merck and used without further purification. All reactions involving oxygen or moisture-sensitive compounds were carried out under dry nitrogen atmosphere. Ice-water and silicon baths were used for reactions at low and high temperatures, respectively, with all reaction temperatures referring to the external bath. Organic extracts were dried over anhydrous Na_2SO_4, filtered and concentrated using a rotary evaporator (Büchi® Rotavapor® R-210, Büchi® B-491 Heating Bath 120V, KNF Neuberger D-79112 Vacuum Pump N 035.1.2 AN.18).

Reactions were monitored by thin-layer chromatography (TLC) using aluminum-backed Merck 60 F_{254} silica gel plates and *n*-hexanes-ethyl acetate solvent systems. After visualization under ultraviolet light at 254 nm and 365 nm, the plates were developed by immersion in a solution containing a mixture of *p*-anisaldehyde (2.5%), acetic acid (1%), and sulfuric acid (3.4%) in 95% ethanol followed by heating. Solid compounds were mixed with SiO_2, redissolved in DCM, and concentrated under reduced pressure before purification through column chromatography using silica gel (Aldrich, 230-400 mesh) and EtOAc-hexanes mixtures. Compounds were systematically named following IUPAC recommendations with ChemDraw v20.0.0.41 (Perkin-Elmer, Waltham, MA, USA).

NMR spectra were recorded in $CDCl_3$ or MeOH-d_4 solutions on a Bruker NMR spectrometer (Bruker Advance III 400 MHz Ascend, 9.4 Tesla), and chemical shifts are reported on the δ scale (ppm) using the residual solvent signals [δ = 7.26 ppm (^{1}H, s, $CDCl_3$); δ = 77.0 ppm (^{13}C, t, $CDCl_3$)] or [δ = 3.31 ppm (^{1}H, qu, MeOH-d_4), 4.78 ppm (^{1}H, s, MeOH-d_4); δ = 49.2 ppm (^{13}C, hep, MeOH-d_4)] as internal standards. Coupling constants (J) are reported in Hz. FT-MS analysis were done on a LTQ OrbitrapTM XL hybrid mass spectrometer (Thermo Fischer Scientific, Bremen, Germany) controlled by LTQ Tune Plus and Xcalibur 2.1.0.

ESI = Electrospray ionization; EtOAc = Ethyl acetate; EtOH = Ethanol; hep = heptet; NBS = *N*-Bromosuccinimide; NMR = Nuclear magnetic resonance; FT-MS = Fourier transform mass spectrometry; qu = quintet; *rt* = Room temperature; s = singlet; t = triplet; THF = Tetrahydrofuran; TLC: Thin layer chromatography.

1.1. *N*-(5-(4-Fluorophenyl)pyrazin-2-yl)acetamide (F-Clmd)

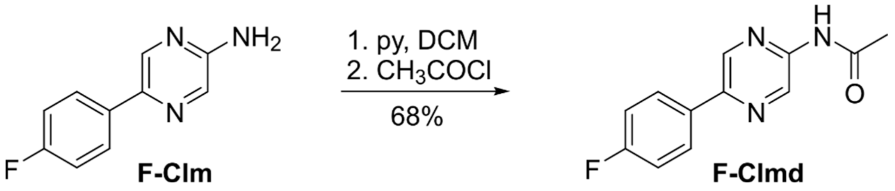

Pyridine (0.056 mL, 0.694 mmol, 1.75 eq) was added to a solution of 5-(4-fluorophenyl)pyrazin-2-amine (**F-Clm**) (0.075 g, 0.396 mmol, 1 eq) in DCM (5 mL), which was previously cooled to 0 ºC, and stirred at that temperature for 5 min. Then acetyl chloride (0.085 mL, 1.189 mmol, 3 eq) was added and the mixture stirred at *rt* until no

starting material was detected by TLC (1:1 EtOAc-hexanes). The reaction mixture was washed with brine and extracted with DCM. The combined organic layers were dried with anhydrous sodium sulfate, filtered, and concentrated under reduced pressure to give a yellowish solid, which was purified by column chromatography (SiO_2, hexanes-EtOAc gradient) to afford *N*-(5-(4-fluorophenyl)pyrazin-2-yl)acetamide (**F-Clmd**) as a white solid [0.063 g, 68%, R_f = 0.39 (50% EtOAc/hex)].

^{1}H NMR (400 MHz, Acetone) δ = 9.76 (bs, 1H), 9.51 – 9.44 (d, *J*=1.6, 1H), 8.88 – 8.76 (d, *J*=1.6, 1H), 8.20 – 8.06 (m, 2H), 7.45 – 7.14 (m, 2H), 2.41 – 2.20 (s, 3H). **^{13}C NMR** (101 MHz, Acetone) δ = 170.0 (C=O), 166.8 – 162.9 (d, *J*=246.8, C-F), 148.7 (C), 147.2 (C), 139.9 (CH), 136.3 (CH), 133.9 (C), 129.3 (CH), 130.2 – 127.4 (d, *J*=8.4, 2xCH), 117.8 – 114.4 (d, *J*=21.8, 2xCH), 24.1 (CH_3). **FTMS-ESI (+)**: m/z: calcd for $[C_{12}H_{11}FN_3O]^+$: 232.0886 $[M+H]^+$; found 232.0885 $[C_{12}H_{11}FN_3O]^+$.

1.2. *N*-(5-(4-Chlorophenyl)pyrazin-2-yl)acetamide (Cl-Clmd)

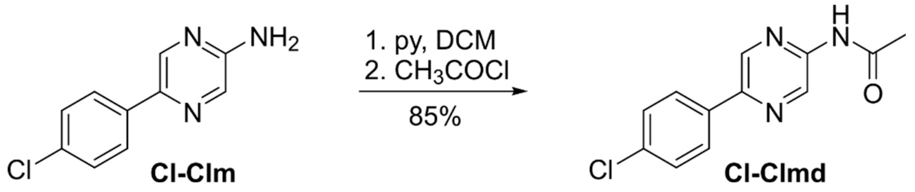

Pyridine (0.069 mL, 0.851 mmol, 1.75 eq) was added to a solution of 5-(4-chlorophenyl)pyrazin-2-amine (**Cl-Clm**) (0.100 g, 0.4863 mmol, 1 eq) in DCM (10 mL), which was previously cooled to 0 ºC, and stirred at that temperature for 5 min. Then acetyl chloride (0.104 mL, 1.459 mmol, 3 eq) was added and the mixture stirred at *rt* until no starting material was detected by TLC (1:1 EtOAc-hexanes). The reaction mixture was washed with brine and extracted with DCM. The combined organic layers were dried with anhydrous sodium sulfate, filtered, and concentrated under reduced pressure to give a yellowish solid, which was purified by column chromatography (SiO_2, hexanes-EtOAc gradient) to afford *N*-(5-(4-chlorophenyl)pyrazin-2-yl)acetamide (**Cl-Clmd**) as a white solid [0.102 g, 85%, R_f = 0.30 (50% EtOAc/hex)].

^{1}H NMR (400 MHz, Acetone) δ = 9.78 (bs, 1H), 9.61 – 9.40 (d, *J*=1.5, 1H), 8.99 – 8.76 (d, *J*=1.5, 1H), 8.29 – 7.97 (m, 2H), 7.67 – 7.32 (m, 2H), 2.25 (s, 3H). **^{13}C NMR** (101 MHz, Acetone) δ = 170.0 (C=O), 149.1 (C), 146.9 (C), 140.2 (CH), 136.4 (CH), 135.6 (C), 130.0 (2xCH), 128.8 (2xCH), 24.2 (CH_3). **FTMS-ESI (+)**: m/z: calcd for $[C_{12}H_{11}ClN_3O]^+$: 248.0591 $[M+H]^+$; found 248.0591 $[C_{12}H_{11}{}^{35}ClN_3O]^+$, 250.0559 $[C_{12}H_{11}{}^{37}ClN_3O]^+$.

---

2. Supporting Figures

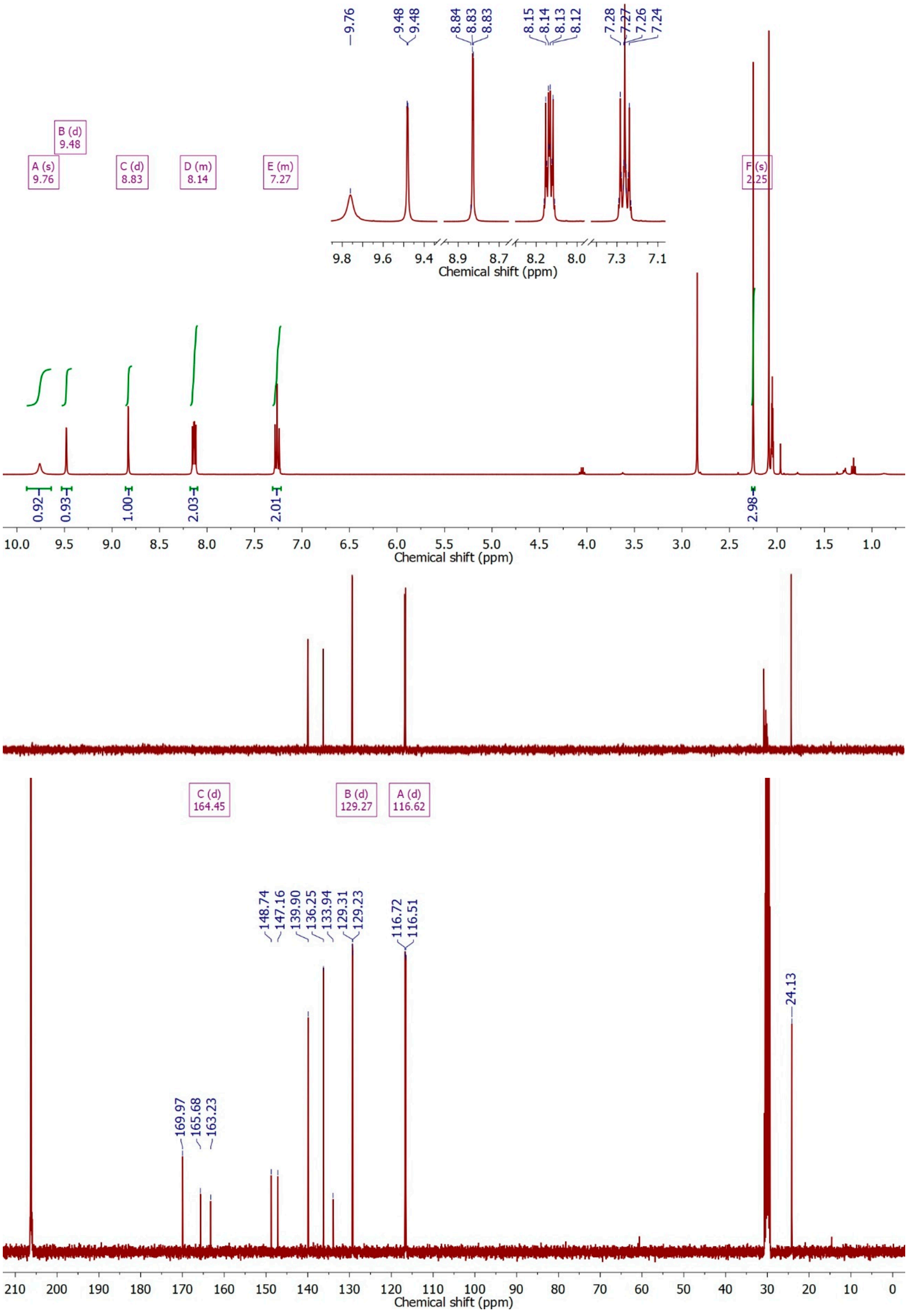

Figure S1. ^{1}H-NMR, ^{13}C-NMR, and DEPT spectra of *N*-(5-(4-fluorophenyl)pyrazin-2-yl)acetamide (**F-Clmd**): ^{1}H NMR (400 MHz, Acetone) δ = 9.76 (bs, 1H), 9.51 – 9.44 (d, *J*=1.6, 1H), 8.88 – 8.76 (d, *J*=1.6, 1H), 8.20 – 8.06 (m, 2H), 7.45 – 7.14 (m, 2H), 2.41 – 2.20 (s, 3H); ^{13}C NMR (101 MHz, Acetone) δ = 170.0 (C=O), 166.8 – 162.9 (d, *J*=246.8, C-F), 148.7 (C), 147.2 (C), 139.9 (CH), 136.3 (CH), 133.9 (C), 129.3 (CH), 130.2 – 127.4 (d, *J*=8.4, 2xCH), 117.8 – 114.4 (d, *J*=21.8, 2xCH), 24.1 (CH_3).

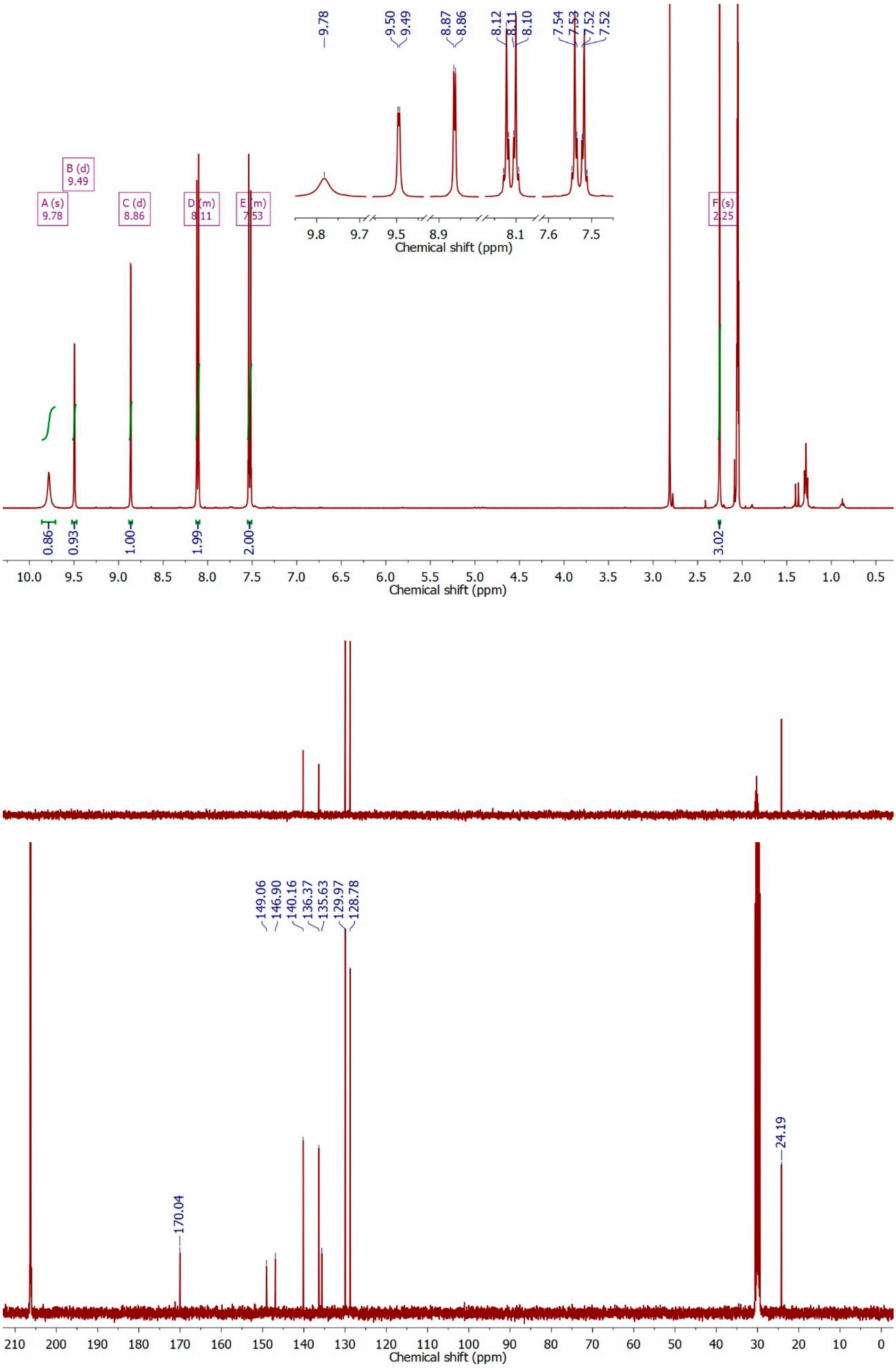

Figure S2. ^{1}H-NMR, ^{13}C-NMR, and DEPT spectra of *N*-(5-(4-chlorophenyl)pyrazin-2-yl)acetamide (**Cl-Clmd**): ^{1}H NMR (400 MHz, Acetone) δ = 9.78 (bs, 1H), 9.61 – 9.40 (d, *J*=1.5, 1H), 8.99 – 8.76 (d, *J*=1.5, 1H), 8.29 – 7.97 (m, 2H), 7.67 – 7.32 (m, 2H), 2.25 (s, 3H); ^{13}C NMR (101 MHz, Acetone) δ = 170.0 (C=O), 149.1 (C), 146.9 (C), 140.2 (CH), 136.4 (CH), 135.6 (C), 130.0 (2xCH), 128.8 (2xCH), 24.2 (CH_3).

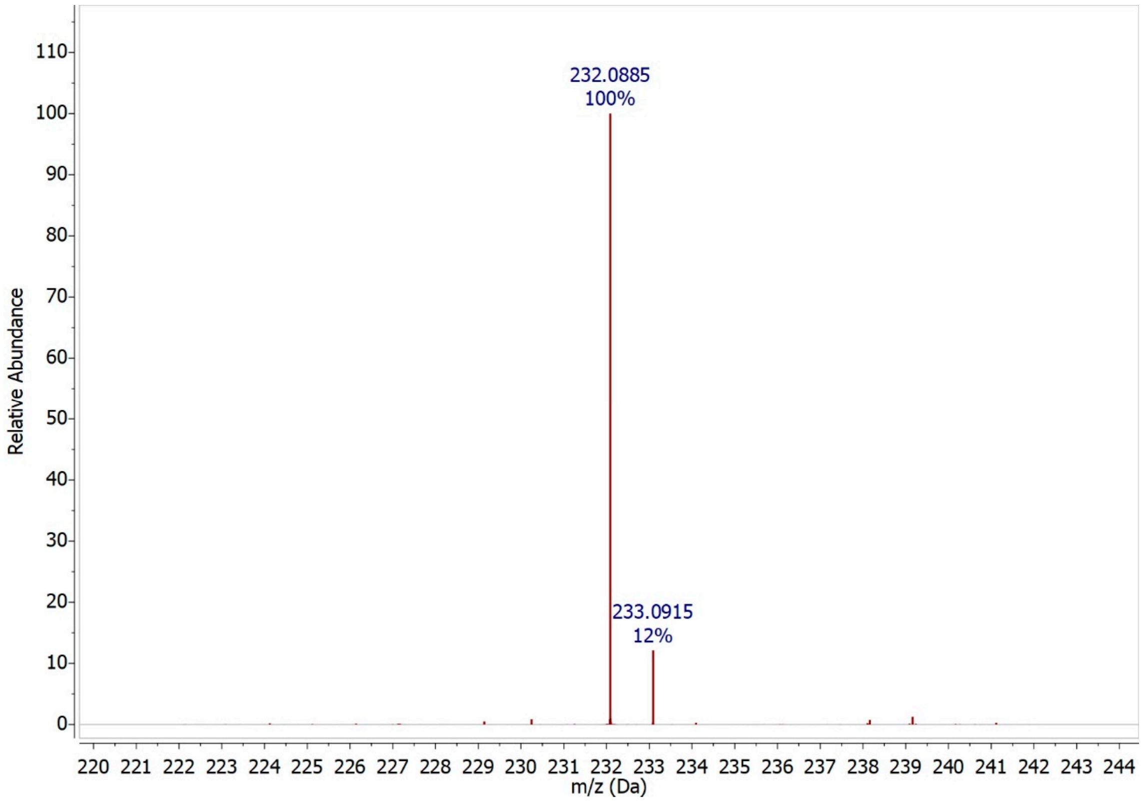

Figure S3. FTMS-ESI (+) spectrum of *N*-(5-(4-fluorophenyl)pyrazin-2-yl)acetamide (**F-Clmd**): m/z: calcd for $[C_{12}H_{11}FN_3O]^+$: 232.0886 $[M+H]^+$; found 232.0885 $[C_{12}H_{11}FN_3O]^+$.

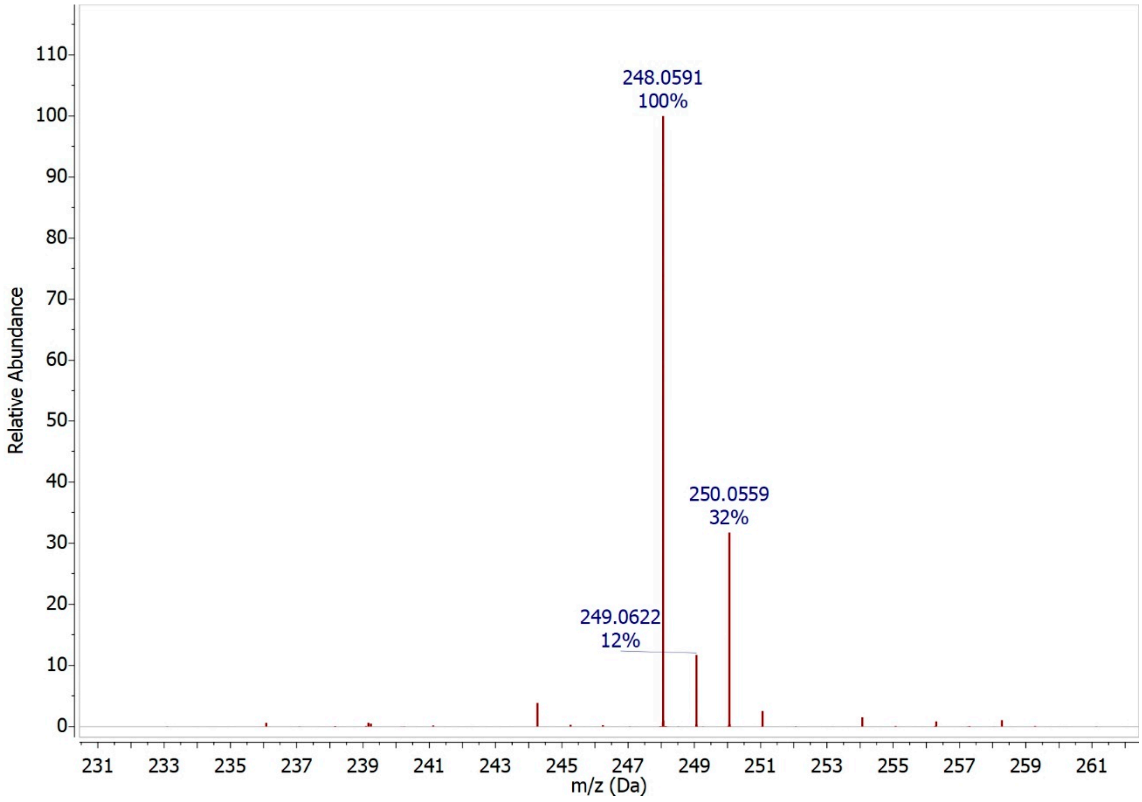

Figure S4. FTMS-ESI (+) spectrum of *N*-(5-(4-chlorophenyl)pyrazin-2-yl)acetamide (**Cl-Clmd**): m/z: calcd for $[C_{12}H_{11}ClN_3O]^+$: 248.0591 $[M+H]^+$; found 248.0591 $[C_{12}H_{11}{}^{35}ClN_3O]^+$, 250.0559 $[C_{12}H_{11}{}^{37}ClN_3O]^+$.
